# Non-Dialysis-Associated Encapsulating Peritoneal Sclerosis: A Unique Case Presentation and Surgical Intervention

**DOI:** 10.7759/cureus.76701

**Published:** 2024-12-31

**Authors:** William R Norman, Selenny Estefany Ricardo Ramirez, Jason R Seale

**Affiliations:** 1 Medicine, Alabama College of Osteopathic Medicine, Dothan, USA; 2 General Practice, Universidad Nacional Pedro Henríquez Ureña (UNPHU), Santo Domingo, DOM; 3 Surgery, Decatur Morgan Hospital, Decatur, USA

**Keywords:** encapsulating peritoneal sclerosis, gastroenterology, general surgery, infectious disease, tuberculosis

## Abstract

Encapsulating peritoneal sclerosis (EPS) is a rare condition involving a thick fibrocollagenous membrane surrounding the small intestine, often associated with peritoneal dialysis. Its occurrence in non-dialysis patients is not well-studied. A man in his 30s from India presented with severe abdominal pain, nausea, vomiting, and constipation. CT imaging revealed small bowel dilation with a transition zone. Diagnostic laparoscopy and exploratory laparotomy identified a thick fibrous rind encasing the small bowel, suspected to be linked to peritoneal tuberculosis (TB) based on positive QuantiFERON-TB and purified protein derivative (PPD) tests. This case highlights that EPS can occur in patients without a history of dialysis, potentially due to TB. Recognition of this rare condition and further research into its causes is essential for improving diagnostic and treatment strategies.

## Introduction

Encapsulating peritoneal sclerosis (EPS) is a rare disease characterized by an acquired thick, fibrous membrane that envelops parts of the small intestine, leading to symptoms of bowel obstruction. This rare disorder is most strongly associated with long-term peritoneal dialysis, which can induce chronic inflammation and eventually lead to sclerosis [1]. However, long-term peritoneal dialysis is not required to develop this condition; this report examines a case where EPS occurred in its absence.

The frequency of EPS varies across the globe between 0.5% and 7.3% but may be as high as 17.2% in patients undergoing PD for 15 or more years [2]. EPS can be classified based on the cause of the inflammatory process into primary or idiopathic and secondary. In the etiology of idiopathic EPS, no specific cause can be identified [3]; in contrast, secondary EPS has been associated with both local and systemic factors that can provoke peritoneal inflammation [4]. These factors include medications [5], infections such as tuberculosis (TB) [6], intraperitoneal mechanical or chemical irritants [7], cirrhosis [8], organ transplantation [9], endometriosis [10], gynecological neoplasms [11], ruptured dermoid cysts [12], and systemic rheumatologic and inflammatory disorders [13] disease. The diagnosis of EPS is primarily based on clinical suspicion, confirmed through radiologic findings such as sclerosis, calcification, peritoneal thickening, or encapsulation of the intestines. Pathologic confirmation is obtained in cases managed surgically [14]. In this case report, we describe the clinical manifestations, evaluation, potential etiologies, and surgical management of this condition. We explore potential secondary causes of EPS, including infections such as TB, and discuss the surgical approach to managing this rare but significant condition.

## Case presentation

On day 1, a male patient in his early 30s, originally from India and living in the United States for two years, had been admitted from the emergency department for a two-day history of severe, persistent abdominal pain, vomiting, and constipation. Upon interviewing him on the medical floor, he reported at least six months of intermittent, similar episodes, though less severe, with symptoms exacerbated by eating and relieved by vomiting. He denied fever, chills, or diarrhea but noted some weight loss. Physical examination revealed mild diffuse abdominal tenderness without rebound or guarding. His vital signs were within normal limits, and he had no significant medical or surgical history.

**Figure 1:**
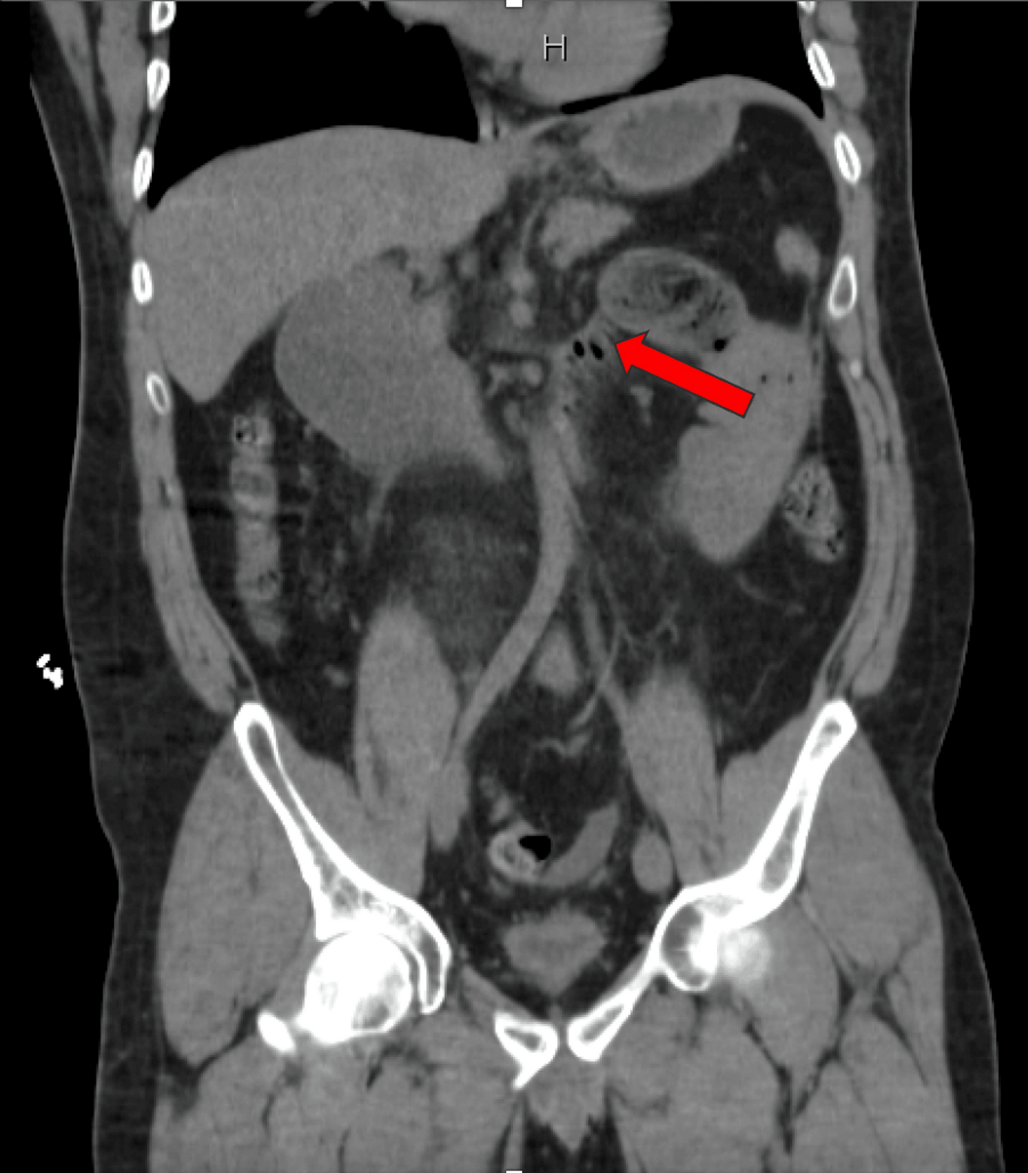
CT scan of abdomen and pelvis showing transition point in the left upper quadrant (denoted by the red arrow)

Initial laboratory workup, including complete blood count (CBC) and comprehensive metabolic panel (CMP), was unremarkable, while urinalysis showed trace protein and ketones. A computed tomography (CT) scan revealed moderate dilation of the proximal small bowel with a transition zone in the left upper quadrant, inspissated material in the distal small bowel, and trace ascites (Figure 1). A nasogastric (NG) tube was placed, and bowel rest was initiated.

**Figure 2:**
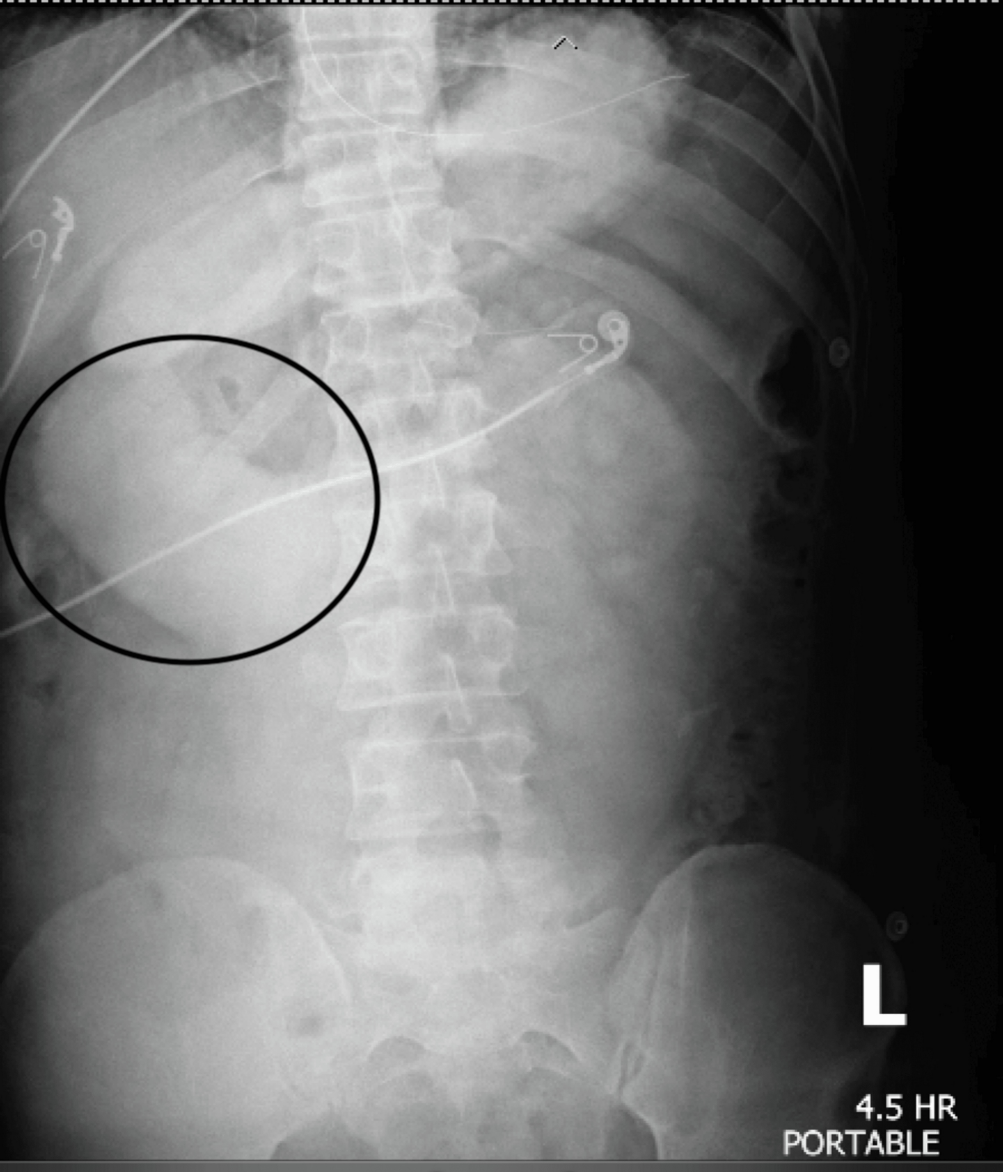
Small bowel pass-through showing moderate diffuse dilation of the duodenum (denoted by the encircled portion) and significantly delayed oral contrast transit time with the contrast column only reaching the distal duodenum after 4.5 hours

On day 2, the patient’s abdominal pain and nausea persisted, with no flatus or bowel movements. Physical examination revealed a somewhat firm and moderately tender abdomen with mild guarding but no rebound tenderness. A small bowel X-ray series (Figure 2) showed contrast reaching only the proximal small bowel after 4.5 hours, with no passage to the colon. Given the findings and persistent symptoms, a diagnostic laparoscopy was planned.

The patient underwent a diagnostic laparoscopy, exploratory laparotomy, and extensive lysis of adhesions, lasting over two hours. Under general anesthesia, with an estimated blood loss of 50 mL, a specimen of the inflammatory rind of the parietal and visceral peritoneum was collected. An extensive inflammatory process encasing the small bowel was observed, adhering to the anterior and lateral peritoneum, parts of the liver lobes, and the sigmoid colon, causing numerous small bowel obstructions from the ligament of Treitz to the terminal ileum. No lymphadenopathy, masses, diverticulitis, appendicitis, or cholecystitis were observed, suggesting a chronic process. The surgical technique included blunt and meticulous dissection to mobilize the small bowel and remove the inflammatory rind, followed by warm saline irrigation, secure closure, and a sterile dressing.

On day 3, the patient reported feeling overall improvement with reduced pain and no nausea, though he had not yet passed gas or had a bowel movement. Physical examination and laboratory data were unremarkable. The plan included continuing the NG tube for bowel rest while monitoring for the return of bowel function. EPS was considered in this case despite the patient’s lack of prior peritoneal dialysis due to the clinical presentation of intestinal obstruction combined with imaging findings suggestive of peritoneal thickening and encapsulation of the intestines. During surgery, the presence of a dense, fibrous membrane encasing the abdominal organs further supported the suspicion of EPS. These findings, in conjunction with the patient’s clinical history and lack of other potential causes such as peritoneal dialysis, pointed toward an alternative etiology.

From days 4 to 12, the patient showed steady improvement. A positive QuantiFERON-TB test and PPD skin test suggested TB as a potential underlying cause of secondary EPS. Follow-up CT imaging showed resolution of the obstruction with passage of contrast into the colon. A CT-guided aspiration of pericolic fluid was performed for culture and TB workup. He denied further nausea, vomiting, or diarrhea, and his abdominal tenderness was limited to the surgical site.

By day 13, the patient tolerated a liquid diet, passed gas, and had a bowel movement. He ambulated well, and his physical examination was unremarkable, with a clean and intact incision. The peritoneal fluid culture was negative for bacterial growth, and the smear for acid-fast bacilli was negative.

On day 14, he was discharged on the rifampin, isoniazid, pyrazinamide, and ethambutol regimen (RIPE regimen) for latent TB, with a discharge diagnosis of suspected EPS secondary to peritoneal TB. Infectious disease was consulted for outpatient follow-up to monitor his progress and response to treatment. The overview of the case is summarized in Table 1.

**Table 1:** Case overview RIPE: rifampin, isoniazid, pyrazinamide, and ethambutol; PPD: purified protein derivative

Category	Key details
Patient background	Male, early 30s, originally from India, living in the United States for two years, presenting with recurrent abdominal pain and recent weight loss.
Symptoms	Severe abdominal pain, vomiting, and constipation lasting two days, preceded by at least six months of intermittent similar symptoms.
Initial imaging	CT showed dilation of the proximal small bowel with an apparent transition zone in the left upper abdomen.
Surgical findings	Extensive inflammation encasing the small bowel, adhering to surrounding organs, causing multiple obstructions.
Key lab findings	Positive QuantiFERON-TB and PPD tests.
Outcome	Discharged on RIPE regimen for latent tuberculosis, scheduled for follow-up.

## Discussion

While EPS is most commonly associated with long-term peritoneal dialysis, our patient had no history of dialysis, making this an atypical presentation. The underlying etiology of EPS in this patient remains unclear, though TB emerged as a possible cause after he tested positive for a QuantiFERON-TB Gold test and PPD skin test, which was substantiated by his immigration history from India, where TB is endemic. However, further diagnostic workup, including a CT-guided aspiration of peritoneal fluid, did not reveal acid-fast bacilli or bacterial growth after one week, leaving peritoneal TB unconfirmed. It should be noted that an extensive number of etiologies have been cited as potential causes of secondary EPS, including abdominal TB, cytomegalovirus peritonitis, granulomatous peritonitis due to parasitic infection, recurrent peritonitis, sarcoidosis, systemic lupus erythematosus, familial Mediterranean fever, and protein S deficiency [15]. This patient underwent serologic testing for blastomyces, coccidioides, cryptococcus, histoplasma, human immunodeficiency virus 1 and 2, cytomegalovirus, A. galactomannan, and beta-(1,3)-D-glucan, all of which were negative. His immunologic testing yielded negative results for antinuclear antibodies and double-stranded DNA antibodies and demonstrated normal complement C3 and C4 levels, lessening suspicion of an autoimmune etiology. Extensive testing for sarcoidosis was not performed, but it should be noted that this patient demonstrated no bilateral hilar lymphadenopathy on chest X-ray and lacked any personal or family history suspicious for this condition. In the absence of prior medical documentation for this patient, it is difficult to determine whether he has a history of recurrent peritonitis. However, this etiology should not be ruled out, especially in the context of positive serology for TB.

It should be noted that EPS occurs in two stages: an inflammatory phase and a fibrotic phase. Corticosteroids are cited to be effective in managing the inflammatory phase, while tamoxifen is cited to inhibit the progression of the fibrotic phase. Ideally, these medications would be administered at the corresponding phases. Surgical intervention is typically reserved for advanced cases with significant fibrosis, where the treatment window has already passed [16]. Although the patient in this case presented at a late stage requiring surgery, with close follow-up, he may avoid future surgeries for recurrent EPS. The recurrence rate for EPS is approximately 20% [17], which could necessitate repeat surgeries. In cases where surgical intervention has already been performed, careful monitoring and a trial of corticosteroids and tamoxifen should be considered to prevent the recurrence of this challenging condition.

## Conclusions

This case highlights the diagnostic challenges and complexities of managing EPS in a patient without a history of peritoneal dialysis, which is traditionally the most recognized cause of EPS. While the patient’s clinical picture and positive TB tests suggested abdominal TB as a potential secondary cause, conclusive evidence was lacking, with negative culture and smear results. This underscores the limitations of current diagnostic methods for peritoneal TB, particularly in cases where imaging and serology suggest but do not confirm an infection. The patient’s favorable response to surgery and his subsequent RIPE regimen indicate that prompt surgical intervention, coupled with targeted antimicrobial therapy, can lead to significant improvement in EPS cases suspected of infectious etiology.

Further studies are needed to better delineate the relationship between EPS and latent infections such as TB, especially in immigrant populations from endemic regions. A more standardized approach to diagnosing secondary EPS, considering both infectious and autoimmune causes, may enhance the accuracy of identifying etiologies and subsequently guide treatment. This case illustrates the importance of a comprehensive workup and individualized care, particularly for patients presenting with atypical EPS profiles, and advocates for continued research into the pathogenesis and optimal management strategies for secondary EPS.
